# Solar Open Flux Migration from Pole to Pole: Magnetic Field Reversal

**DOI:** 10.1038/s41598-017-09862-2

**Published:** 2017-08-25

**Authors:** G.-H. Huang, C.-H. Lin, L. C. Lee

**Affiliations:** 1 Graduate Institute of Space Science, National Central University, Taoyuan, Taiwan; 2 Institute of Earth Sciences, Academia Sinica, Taipei, Taiwan

## Abstract

Coronal holes are solar regions with low soft X-ray or low extreme ultraviolet intensities. The magnetic fields from coronal holes extend far away from the Sun, and thus they are identified as regions with open magnetic field lines. Coronal holes are concentrated in the polar regions during the sunspot minimum phase, and spread to lower latitude during the rising phase of solar activity. In this work, we identify coronal holes with outward and inward open magnetic fluxes being in the opposite poles during solar quiet period. We find that during the sunspot rising phase, the outward and inward open fluxes perform pole-to-pole trans-equatorial migrations in opposite directions. The migration of the open fluxes consists of three parts: open flux areas migrating across the equator, new open flux areas generated in the low latitude and migrating poleward, and new open flux areas locally generated in the polar region. All three components contribute to the reversal of magnetic polarity. The percentage of contribution from each component is different for different solar cycle. Our results also show that the sunspot number is positively correlated with the lower-latitude open magnetic flux area, but negatively correlated with the total open flux area.

## Introduction

Coronal holes are often observed as the regions with low optical intensity (LOI) in soft X-ray or extreme ultraviolet (EUV) images. They are found to locate inside regions that are of weak and predominantly unipolar magnetic field (Altschuler *et al*.^1^). Theoretically, the coronal holes are defined as the regions in which the magnetic field lines extend far away from the Sun (e.g., Obridko & Shelting^2^ and the references therein), in other words, the regions with open magnetic field lines (OMF).

Based on their distribution on the solar surface, coronal holes are often divided into polar and non-polar coronal holes (Sanchez-Ibarra & Barraza-Paredes^3^). Polar coronal holes (PCHs) are mostly seen above latitude 65° during the sunspot minimum phase. Despite their name, PCHs are not restricted to polar regions, but can extend to lower latitudes and even the other hemisphere during the decreasing phase of solar activity. The PCHs are observed to have quasi-rigid rotation^4–7^. In contrast, non-polar coronal holes (or equatorial coronal holes) mostly appear within latitude ±30° during solar maximum phase, and show significant differential rotation^6–8^.

Since coronal holes are predominantly unipolar regions on the Sun, they are good tracers of the change of the solar magnetic-field polarities, and many works have been conducted to examine the variations of different properties of coronal holes. The evolution of the number and locations of coronal holes was found to be correlated with solar magnetic fields (Bilenko^9^). Hess Webber *et al*.^10^ measured the area of the polar coronal holes over 1996–2010, and found that the areas in the two hemispheres did not reach maximum at the same time. Karna *et al*.^11^ investigated the relationship between the area of polar coronal holes and solar magnetic fields over solar cycle 24, and found inverse correlation between the area of PCH and sunspot number. Their results also showed connection from the magnetic fields of active regions to those of the polar regions. Karachik *et al*.^12^ reported the formation of two non-polar coronal holes from the dissipation of the magnetic fields of four active regions. Ikhsanov & Ivanov^8^ analyzed different properties of equatorial and polar coronal holes from 1970 to 1995, including numbers, locations, areas and polarities, and reported that both types of coronal holes can be further divided into multiple sub-classes based on the temporal variation of their properties. A recent study by Bilenko & Tavastsherna^13^ examined the evolution of coronal holes and solar global magnetic fields over three solar cycles from 1976 to 2012. By analyzing the temporal variation of coronal hole location, they reported that non-polar coronal holes exhibit a poleward motion and a sinusoidal motion. These studies have all identified coronal holes as the dark regions in EUV or X-ray images (i.e., LOI coronal holes). Obridko & Shelting^2^ pointed out that LOI coronal holes and open field regions are not completely identical physical objects although the two are often associated with each other in statistical sense. They studied the evolution of open field regions from 1970 to 1996. Their comparison between the sunspot locations and open flux ratio (i.e., open flux/total flux) showed a close relationship between mid-latitude coronal holes and active regions.

In this work, we first compared the LOI and OMF coronal holes with source regions of high-speed solar wind events, and found that the LOI and OMF coronal holes do not always coincide and that high-speed solar wind events are more likely to originate from OMF coronal holes than from LOI coronal holes. We then examined the temporal variations of the area and magnetic polarities of OMF coronal holes at different latitudes over three and half solar cycles from 1976 to 2014.

## Identification of coronal holes

Coronal holes have generally been identified as regions of either low optical intensity (LOI coronal holes) or open magnetic field lines (OMF coronal holes). Since coronal holes (CHs) are major source regions of high-speed solar wind streams (HSSs)^14, 15^, which can cause significant geomagnetic activity at Earth^16^, we first examine whether one method may be better than the other to identify the source regions of HSSs. To identify LOI coronal holes, solar images from the Atmospheric Imaging Assembly (AIA) 193 Å and radial field magnetograms from Helioseismic Magnetic Imager (HMI) onboard the Solar Dynamic Observatory (SDO) were used to create synoptic maps. The time period of the AIA and HMI data is from June 2010 to December 2014, corresponding to Carrington rotation number 2099 to 2158. The LOI CHs were identified from the synoptic maps based on the procedures described by Krista & Gallagher^17^. To identify OMF coronal holes, we used the radial-field synoptic maps from the Wilcox Solar Observatory (WSO) from May 1970 to December 2014, corresponding to Carrington rotation number 1642 to 2158. Each synoptic map consists of 60 by 30 pixels in longitude (*ϕ*) and sine-latitude (sin *λ*) coordinate, resulting in a spatial resolution of Δ*ϕ* = 2*π*/60 in longitudinal direction, and Δ(sin *λ*) = 2/30 in sine-latitude. The area of a pixel in the map thus equals to *r*Δ*ϕ*Δ(sin *λ*), and is same at all latitudes. To determine whether the field line of a pixel would extend to infinity, we need to first know the three-dimensional magnetic field above the solar surface. Earlier studies on the magnetic field above the photosphere indicate that the inner corona can be considered current free (Schatten *et al*.^18^) and that the current is only significant during rapid development of active region (Harvey^19^). Since the magnetic field structure of interest in our study is the large-scale global fields that do not change over one solar rotation, the structure can be assumed to be current free, and a Potential Field Source Surface (PFSS) model^18^ was applied to the synoptic map to construct the 3-D magnetic field. The constructed magnetic field is current free between the solar surface and an upper boundary (source surface), where all field lines become radial. In this work, the source surface is placed at $2.5\,{{\rm{R}}}_{\odot }$ from the solar center, following earlier studies (e.g., Wang & Sheeley^20^, Obridko & Shelting^2^). After the 3D magnetic fields were constructed, the field lines were traced from the source surface to the solar surface, and the footpoints of the open magnetic field lines on the solar surface were identified as OMF CHs.

The solar wind speed data were obtained from the Solar Wind Electron Proton Alpha Monitor (SWEPAM) onboard the Advanced Composition Explorer (ACE) from June 2010 to December 2014. We followed the criteria described by Xystouris *et al*.^21^ to identify HSS events, and excluded the events that may be caused by solar flare-related coronal mass ejections by examining the X-ray data from the GOES-12 satellite. A total number of 118 high-speed solar wind stream events were selected.

To determine the source longitudes of the HSS events, we need to trace the trajectories of the HSSs back to the solar surface. The exact trajectory and velocity profile of HSS through the interplanetary space are currently not observable. As a first order approximation, we assumed that the solar wind is propagating radially at constant speed (Parker^22^). This assumption is based on the fact that HSS is moving at a speed much higher than the Alfvén speed and sound speed in the interplanetary space and that *in*-*situ* measurements of solar wind at 1 AU often show that the radial velocity is much higher than the transverse components. We caution that this simplistic assumption inevitably introduced some errors to the determined source longitudes.

We considered a CH to be the source of an HSS event if the source longitude of the HSS is located within the CH boundary. If the source longitude passes two or more CHs, we selected the one located at a lower latitude because the solar wind from a lower latitude CH would be more likely to reach 1 AU in the equatorial plane. If the source longitude of an event is within an OMF region and an LOI region, both regions are considered as the source. Out of the 118 HSS events, 111 events can be traced to OMF regions, 63 events can be traced to LOI regions, and 6 events cannot be mapped to any identified CHs. Among the OMF coronal holes that were the source regions of the HSS events, 48 of them were also LOI coronal holes. There were 49 events that only hit OMF coronal holes, and only 1 event that only hit LOI coronal hole. There were also 14 events whose souce longitudes pass an OMF CH and an LOI CH, but the two coronal holes do not coincide.

Overall, the analysis indicates that OMF CHs and LOI CHs do not always coincide with each other, and that the OMF CHs are more likely to be the source of HSS signals. For the rest of this paper, the term “coronal hole” will be interchangeable with “open magnetic flux region”.

## Solar cycle variations of coronal holes

**Figure 1:**
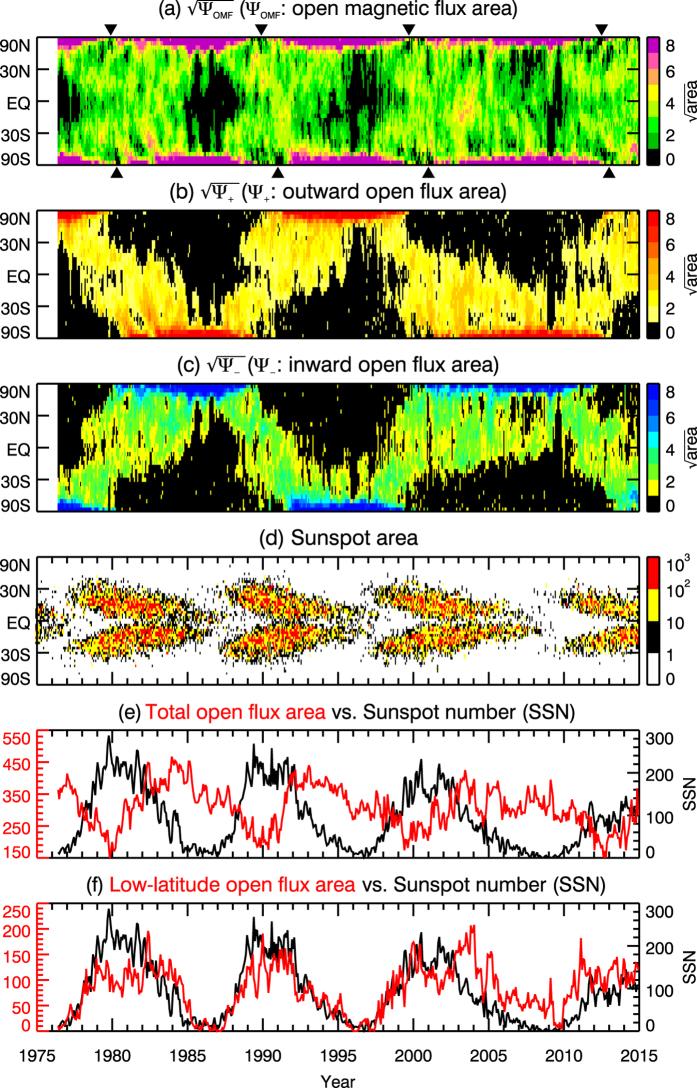
The upper three panels are the time maps of (**a**) open magnetic flux, (**b**) outward open magnetic flux, and (**c**) inward open magnetic flux. The lower three panels show (**d**) the sunspot butterfly diagram, (**e**) the total open magnetic flux area versus sunspot number (SSN), and (**f**) the low-latitude open magnetic flux area versus the SSN. The open flux area is in units of pixel (1 pixel = *r*Δ*ϕ*Δ(sin *λ*)), and the sunspot area is in units of one millionth of the surface area of solar hemisphere. All parameters are plotted as a function of time from 1976 to 2014, and follows the horizontal axis of the bottom panel.

To study the temporal variation of the polarities and area of the CHs at different latitudes, we constructed the unsigned, outward, and inward open magnetic flux map (Ψ_OMF_(*λ*,*t*), Ψ_+_(*λ*,*t*), and Ψ_−_(*λ*,*t*), where Ψ_OMF_ = Ψ_+_ + Ψ_−_) by summing the number of pixels of unsigned, outward, and inward open magnetic field over longitude in each Carrington rotation (*i*.*e*., solar rotation) *t*. The result is plotted in the first three panels of Fig. 1. To enhance the visibility of pattern, the plotted images are $\sqrt{{{\rm{\Psi }}}_{{\rm{OMF}}}(\lambda ,t)}$, $\sqrt{{{\rm{\Psi }}}_{+}(\lambda ,t)}$, and $\sqrt{{{\rm{\Psi }}}_{-}(\lambda ,t)}$. Since there are 60 longitudinal pixels along each latitude stripe *λ*_*j*_, the maximum values of $\sqrt{{{\rm{\Psi }}}_{{\rm{OMF}}}}$, $\sqrt{{{\rm{\Psi }}}_{+}}$, and $\sqrt{{{\rm{\Psi }}}_{-}}$ is $\sqrt{60}$. For brevity, the unsigned open magnetic flux map will be hereafter referred to as open magnetic flux map. To compare the the solar cycle variation of the open magnetic flux area with that of the sunspots, the time map of sunspot area (the so-called “butterfly diagram”) is plotted in Fig. 1(d), and the sunspot number is compared with the total and low-latitude OMF areas in Fig. 1(e,f), respectively. The sunspot area map was constructed from the data from the Royal Greenwich Observatory.

Figure 1(a) shows that the variation of the open magnetic flux area is generally symmetric at the two poles. Figure 1(b,c) reveal a pole-to-pole trans-equatorial pattern over solar cycle. Specifically, the figures show that the outward and inward fluxes are mostly concentrated in the opposite polar regions (>65°) during the quiet period, spread to lower latitude during the rising phase of solar activity, cross the equator around the solar maximum, and reach the opposite pole during the decreasing phase of sunspot number. Such pole-to-pole trans-equatorial migration (PPTE) contributes to the polarity reversal in the two hemispheres. The average migration rate of the outward and inward open magnetic flux is estimated to be approximately 26.7 ± 6.4 deg/year, equivalent to 10.3 ± 2.5 m/s. This is smaller than the measured poleward meridional flow speed at the solar surface, which is approximately 15–20 m s^−1^ (e.g., Duvall^23^).

**Figure 2:**
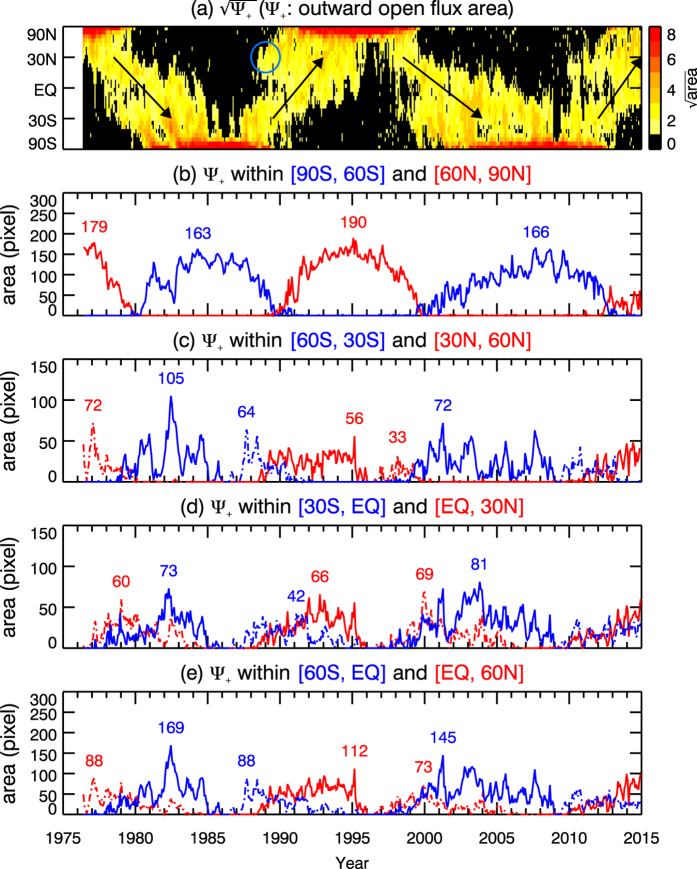
Comparison of the temporal variation of the total outward open flux area in different latitude ranges. Panel (**a**) is the time map of the outward open magnetic flux area. The black arrows indicate the direction of solar cycle evolution. The blue circle marks an example of locally generated open flux area. Total area Ψ_+_ in different latitudinal ranges as a function of time are plotted in panels (**b**)–(**e**). The specific latitudinal ranges are indicated above the corresponding panels. In panels (**b**)–(**e**), red and blue curves represent northern and southern hemispheres, respectively. The dashed and solid lines are to distinguish the curves before and after crossing the equator in each solar cycle. The maximum total area in each hemisphere in each solar cycle is printed above the corresponding peak. The unit of the area is pixel (1 pixel = *r*Δ*ϕ*Δ(sin *λ*)).

To better understand the cause of the PPTE migration pattern, we use the outward open flux area as an example, and plotted the total area in different latitudinal ranges in Fig. 2(b)–(e). The specific latitudinal ranges are 60° to 90°, 30° to 60°, 0° to 30°, and 0° to 60°, as indicated above the corresponding panels. In this discussion, 60° to 90° is considered as the polar region. The time map of the outward open magnetic flux is placed in the top panel of Fig. 2 for comparison. The black arrows are to indicate the temporal direction of solar cycle evolution. In panels (b)–(e), red and blue curves represent the northern and southern hemispheres, respectively. Dashed and solid lines are to distinguish whether the curve is before or after crossing the equator in each solar cycle. The maximum area in each solar cycle in each hemisphere is printed above the corresponding peak. The unit of area in the plot is pixel (1 pixel = *r*Δ*ϕ*Δ(sin *λ*)).

Comparing panels (b) and (c), we can see that the maximum areas in the polar region are larger than those in [30°, 60°] latitude range for all three solar cycles, indicating that some open flux areas in the polar region are locally generated. The difference between the maximum areas in the polar region and in the [30°, 60°] region indicate that the amount of open flux area locally generated at the polar region in each solar cycle is at least 36%, 70% and 57%, respectively, of the total open flux area in the polar region. Therefore, except for the first cycle, majority of the polar region flux is locally generated. For the first cycle, the migration of the open flux from lower latitude to polar region can be clearly seen in Fig. 2(a). There is a stripe (at the tip of the arrow) extending from near equator to the polar region. The small gap in the polar region just before the stripe reaches the polar region coincides the sudden dip in the first cycle in panel (b), and the peak in the first cycle in panel (c) corresponds to the area of the stripe when it is in [30°, 60°]. The addition of the open flux area of this stripe significantly increases the polar region open flux area of this cycle.

Applying similar analysis to the comparison between 30°–60° (panel c) and 0°–30° (panel d), we can see that in first cycle the maximum area in 30°–60° is larger that that in 0°–30°, indicating some new open flux have been generated in the latitude between 30° and 60°. The amount of the locally generated open flux is at least 30% of the total open flux in 30°–60°. In contrast, the maximum area in 30°–60° is lower than that in 0°–30° for the next two cycles, indicating that some open flux in 0°–30° have dissipated locally. The difference indicates that the amount of dissipation is at least 15% and 11% for the second and third cycle, respectively. The maximum amount of open flux that can migrate from 0°–30° to 30°–60° in these two cycles can be estimated as the maximum area in 30°–60°. In short, the maximum amount of migrated open flux area is 73, 56, and 72 pixels.

In panel (d), comparison between the dashed (before crossing the equator) and solid lines (after crossing the equator) show that the peak areas in all three cycles are larger after crossing, indicating that new open fluxes have been generated after crossing the equator. The open fluxes locally generated between the equator and 30° after crossing the equator are at least 18%, 36% and 15% for the three cycles.

In summary, our analysis indicates that the PPTE pattern consists of four components: (1) open fluxes migrating across the equator; (2) locally generated open fluxes dissipating locally without migrating to higher latitudes; (3) locally generated open fluxes migrating to higher latitudes; and (4) open flux locally generated in the polar region. The contribution from each component differ from cycle to cycle. The situation for the inward open flux is expected to be similar except that the direction of migration is opposite.

In contrast to the PPTE migration of the open magnetic flux, sunspots are rarely present at the equator or at latitude higher than ≈35°. This is because the sunspots are formed by the stretching and enhancing of magnetic field lines by solar differential rotation, the latter peaks at the mid latitude but is minimum at the equator and at high latitudes.

In a short period approaching the maximum phase of sunspot number, open flux at the two poles almost completely disappear (indicated by the black triangles in Fig. 1a), leading to a minimum value in the total open flux area (cf. red line in Fig. 1e). Since these points coincide the beginning of polarity reversal (cf. Fig. 1b,c), the complete disappearance of open flux can be explained as the result of zero-point crossing as the magnetic field is changing from one polarity to the other.

This phenomenon was first reported by Waldmeier^24^ in his examination of coronal holes over four solar cycles (1940–1978), and can also be seen in the results of later observational studies (e.g., Ikhsanov & Ivanov^8^ Hess Webber *et al*.^10^).

**Figure 3:**
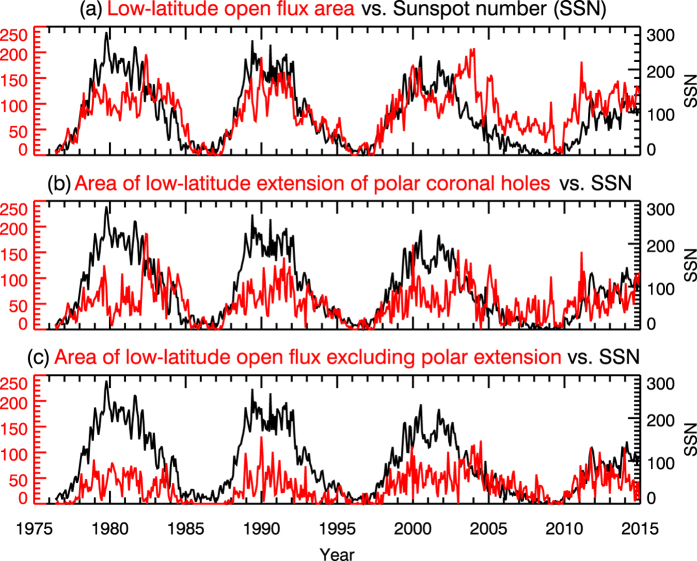
Comparison between the sunspot number profile and the profiles of low-latitude open flux area (**a**), the area of the low-latitude extension of polar coronal holes (**b**) and the area of the low-latitude open flux excluding the polar extension (**c**). The SSN profile is plotted in black and follows the black Y axis on the right hand side. The red lines are different open flux areas, as indicated in the title of respective panel, and follow the red Y axis on the left hand side.

Figure 1(e) shows that there is a general negative correlation between the sunspot number and the total open flux area. The correlation coefficients in solar cycle 21, 22, and 23 are −0.70, −0.62, and −0.52 respectively, with time lags of 0.97, 1.05, and 2.39 years relative to the sunspot number. In contrast, the sunspot number is positively correlated with the open flux area within ±30° latitude, as shown in Fig. 1(f). The correlation coefficients in solar cycle 21, 22, and 23 are 0.62, 0.89 and 0.57 respectively, with 0.0, 0.0, and 0.52 years preceding the sunspot number. The good correlation between SSN and the low-latitude open flux area appears to be broken from the descending phase of Cycle 23. The anomaly in cycle 23 has also been reported by multiple earlier studies (e.g. Hess Webber *et al*.^10^, Karna *et al*.^11^). As described in the introduction, some polar coronal holes can extend to low latitude and even the other hemisphere during the decreasing phase of solar activity. Therefore, the low-latitude open flux area in Fig. 1(f) includes contribution from the low-latitude extensions of polar coronal holes. As an attempt to investigate what causes the disruption of the good correlation in Fig. 1(f), we extracted the contribution from the low-latitude extension of polar coronal holes. In Fig. 3, Fig. 1(f) is replotted in the top panel for comparison, the contribution from the low-latitude extension of polar coronal holes is plotted in the middle panel, and the low-latitude open flux excluding the contribution from the polar extension is plotted in the lower panel. In other words, the coronal hole area in Fig. 3a equals to the sum of the areas in Fig. 3b,c. Figure 3(b,c) shows that both components by themselves are in good correlation with SSN profile for all cycles. However, during the descending phase of cycle 23, both did not decrease as fast as in the previous cycles, leading to larger low-latitude open flux area and the apparent poor correlation with the SSN profile.

## Discussion and Conclusion

Many current solar dynamo models are based on Babcock-Leighton mechanism^25, 26^. The basic idea of the mechanism is that the decay of the tilt of sunspot groups generates the poloidal field of the Sun. The Babcock-Leighton model has since been developed into a range of more elaborated dynamo models (e.g., refs 20, 27–30). For instance, the flux transport model (Wang & Sheeley^20^, Wang^29^) is based on the assumption that the polar open fluxes come from the edge of active regions in the lower latitudes. By including the effects of differential rotation, magnetic diffusion, and meridional flow for the formation of polar open fluxes, the model demonstrated that part of the leading-polarity fluxes of the active regions diffuse to the other hemisphere, while the remaining fluxes are carried pole-ward by the meridional flow. A different model based on observational results was proposed by Ikhsanov & Tavastsherna^31^ and Bilenko & Tavastsherna^13^. They suggested that the polar open fluxes are generated by the poleward transportation of the open fluxes at around 35° latitude.

These models may partly explain the poleward migration of the open flux, but cannot explain the pole-to-equator portion of the migration as shown in Fig. 1(b,c). Based on our results, we propose that the evolution of the outward and inward open magnetic flux areas and polarity reversal of the Sun are associated with a pole-to-pole trans-equatorial (PPTE) migration, which consists of trans-equatorial migration, poleward migration of new flux generated from lower latitudes, and the new flux locally generated at the polar regions. The present results can provide important observational constraints to the solar dynamo models. Specifically, a valid dynamo theory must reproduce the observed PPTE migration pattern of open flux area and the negative (positive) correlation between the sunspot cycle and the total (low-latitude) open flux area. Once a valid model is found, the model can improve our understanding of the dynamo mechanism inside the Sun.
